# PARIETAL PERITONEUM GRAFT FOR DUODENUM INJURIES IN AN ANIMAL MODEL

**DOI:** 10.1590/0102-672020180001e1418

**Published:** 2019-02-07

**Authors:** Joana M. CASTILLO, Anibal FLORES-PLASCENCIA, Maria Delia PEREZ-MONTIEL, Salma GARCIA, Neydel VERGARA, Aida PEREZ-BLANCO, Enrique Alejandro SANCHEZ-VALDIVIESO

**Affiliations:** 1 Department of Surgery, Hospital de Alta Especialidad, Veracruz City, Mexico;; 2 Department of Research, Cristobal Colon University School of Medicine;; 3 Department of Pathology, National Cancer Institute of Mexico, Mexico City, Mexico.

**Keywords:** Peritoneu, Graf, Duodenu, Injur, Repai, Animal mode, Peritôni, Enxert, Duoden, Lesã, Reparaçã, Modelo animal

## Abstract

**Background:**

Duodenal injuries and their surgical procedure cause a high morbidity and mortality.

**Aim:**

To assess the overall effectiveness of the auto-graft of peritoneum in the treatment of the perforation of the duodenum, aiming to reduce surgery time, costs, complexity and mortality.

**Methods:**

Twelve New Zealand rabbits, ages 4-6 months, both sexes, underwent designed surgical grade III duodenal injuries that were repaired 18 h after. Rabbits were surgically treated with the proposed auto-graft of peritoneum.

**Results:**

No postoperative deaths were observed; the animals presented corporal weight increase and were euthanized six months later. There was no significant difference between both groups relating to the postoperative evolution or in the histological changes.

**Conclusion:**

Auto-graft of the peritoneum and posterior fascia is a useful option for duodenal repair and that is worth of evaluation for humans.

## RESUMO

**Racional:**

Lesões duodenais e seu procedimento cirúrgico causam alta morbimortalidade.

**Objetivo:**

Avaliar a eficácia geral de retalho peritoneal no tratamento da perfuração do duodeno, visando reduzir o tempo, os custos, a complexidade e a mortalidade cirúrgicas.

**Métodos:**

Doze coelhos da raça Nova Zelândia, com idades entre 4-6 meses, ambos os sexos, foram submetidos a lesões duodenais cirúrgicas de grau III, que foram reparadas 18 h depois. Coelhos foram tratados cirurgicamente com a proposta de auto-enxerto de peritônio.

**Resultados:**

Não foram observados óbitos pós-operatórios; os animais apresentaram aumento de peso corporal e foram eutanasiados seis meses depois. Não houve diferença significativa entre os dois grupos em relação à evolução pós-operatória ou nas alterações histológicas.

**Conclusão:**

A auto-enxertia do peritônio e da fáscia posterior é uma opção útil para o reparo duodenal e vale a pena ser avaliada em seres humanos.

## INTRODUCTION

The retroperitoneal location of the duodenum plays an important role for being traumatism protected^6^. The incidence of duodenal injuries varies between 3.7% and 5.0%^3^ ^,^ ^23^. Of these injuries, 77.7% are due to penetrating trauma and 22.3% secondary to blunt trauma^2^ ^,^ ^17^. Isolated injuries of the duodenum are not frequent, it is necessary to always keep present the high frequency of associated injuries; this is due to the closeness to mayor vascular structures^5^. Thus, the duodenum is the third most affected digestive structure by blunt trauma, preceded by injuries in jejunum-ileum and colon-rectum^18^ for which it´s recognition and early treatment is important^21^. With blunt trauma, a direct force applied on the abdominal wall is transmitted to the duodenum, which is projected backward against the rigid vertebra, common in sport injuries or car accidents^10^ ^,^ ^12^. Both duodenal lesions and surgical procedure entail high morbidity and mortality^12^ derived from complications for the procedure itself or the formation of a duodenal fistula. 

The peritoneum consists of a monolayer of mesothelium cells lying on a basement membrane covering an area of approximately two square meters in an adult person^11^ ^,^ ^25^. An experimental surgical technique taking a graft of peritoneum might be used for duodenal fistula prevention. 

The aim of this study was to describe the efficacy of placement of this graft as a new surgical technique for duodenal perforation, reducing surgical time, costs, postoperative time of recovery, the rate of complications and mortality.

## METHODS

This study was conducted at the Cristobal Colon University School of Medicine, previous authorization by the Ethics Committee. Adult New Zealand rabbits (*Oryctolagus cuniculus*), 4-6 months of age, of either sex were used in this study. An animal model for grade III duodenal injury was designed, according to the American Association for the Surgery of Trauma-Organ Injury Scale (AAST-OIS, Table 1)^21^. All procedures were carried on with strict attachment to the technical specifications for experimentation in laboratory animals according to the Official Mexican Rules^13^ ^,^ ^22^. 

**TABLE 1:** Duodenum organ injury scale according to the American Association for the Surgery of Trauma

Grade		Injury description
I	Hematoma Laceration	Involving single portion of duodenum Partial thickness, no perforation
II	Hematoma Laceration	Involving more than one portion Disruption < 50% of circumference
III	Laceration	Disruption 50-75% of circumference of D2 Disruption 50-100% of circumference of D1, D3, D4
IV	Laceration	Disruption > 75% of circumference of D2 Involving ampulla or distal common bile duct
V	Laceration Vascular	Massive disruption of duodenopancreatic complex Devascularization of duodenum

D1=1^st^ portion; D2=2^nd^ portion; D3=3^rd^ portion; D4: 4^th^ portion of duodenum^21^

All animals received ketamine (35 mg/kg IM; Anesket, Pisa) and xylazine (5 mg/kg IM; Tranquived, VEDCo). Anesthesia for 30-45 min was achieved by IM injection in the posterior region of the thigh with a 23 Gx1 needle, using ketamine combined with xylazine in the same syringe. After median laparotomy the antimesenteric surface of the duodenum was exposed and a 7 mm^2^ defect, resembling grade III duodenal injury, was created with surgical scissors; the duodenal defect was left open and the abdominal wall was immediately closed. Eighteen hours later, the animal was re-operated, the duodenal perforation identified, and resection of the damaged edges performed leaving a final defect of about 8-9 mm^2^. The injury site was then repaired by the following technique: a sheet of tissue of about 10x15 mm, composed of parietal peritoneum attached to the posterior fascia of the rectus abdominis muscle was obtained^4^. The graft was sutured on as a patch with 5-0 monofilament polypropylene (Prolene, Ethicon, 1/2 circle atraumatic needle), with or without BioGlue^®^ support, with the peritoneal membrane facing and covering the defect; subsequently, the omentum was approached on the graft site contributing to vascular support and the abdominal cavity closed. 

All rabbits were housed in a standard laboratory animal environment (fresh filtered air, 15 changes per hour; temperature, 21±2° C; humidity, 50±20%; and 12:12-h light: dark cycle) and kept in continuous monitoring during six months; afterwards, euthanasia was performed through intra-cardiac administration of 250 mg/kg sodium pentobarbital.

Paraffin-embedded tissue sections were H&E stained. Inflammatory reaction was graded in a 0 to 3 scale (0: no inflammation, 1: mild, 2: moderate, 3: severe inflammation). The repair process was evaluated for the presence of mucosa, muscularis propria and serosa. Immunohistochemical staining was performed on paraffin-embedded tissue sections, using an avidin-streptavidin method.

### Statistical analysis

The data were recorded in Excel^®^; values are expressed as mean+/-standard deviation. Data were analyzed by using ANOVA, and the Mann-Whitney U test was used for the analysis of histopathology data.

## RESULTS

Twelve adult New Zealand rabbits (*Oryctolagus cuniculus*) weighting 2-3.1 kg (median 2.5±0.5 kg) with optimal health and normal level of activity were included. To mimic the clinical situation, we made every effort in order to achieve a standard duodenal defect of 7 mm in diameter during the duodenal injury step.

**FIGURE 1:**
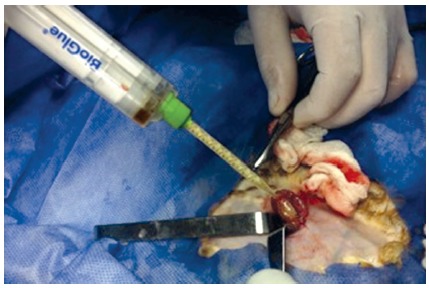
The procedure of making and suturing the graft and application of the Bioglue support is shown

All late (second) procedures were performed without complications; mean surgical time during the 1^st^ operation was 20.1 min and 59 min during the 2^nd^, with a mean time for obtaining and repairing of the peritoneal graft of 18.6±2.5 min (range: 15-20 min). Animals tolerated well the procedure (Figure 1) and there were no immediate postoperative complications.

Oral feeding and ad libitum water were initiated immediately after post-anesthetic care. After recovering from the anesthetic effect they returned to normal activity (eating, drinking water and proper motion) within two days. Postoperative analgesia was provided by ketorolac and quinolone coverage was administered for one week. No postoperative deaths were observed.

With increasing time of follow-up animals gained weight. No biochemical abnormalities were observed. Wound healing variables, recovery time and integration to normal activities, complications, and histopathology reports, were registered and analyzed. The efficiency of the injury repair procedure was also evaluated in terms of peri-operative complications.

Animals were followed-up for six months and, afterwards, euthanasia was achieved through a 250-mg/kg intra-cardiac sodium pentobarbital administration. At the necropsy, no leakage was observed around the duodenal patch. Restitution of the integrity of the intestinal wall was observed in both groups showing the smooth muscle layer lining the duodenal mucosa, with thin compact villi and a light lymphocyte infiltration.

**FIGURE 2:**
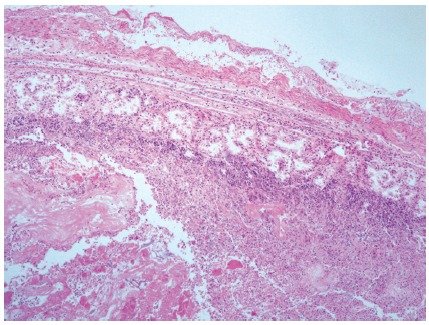
Photomicrograph showing fibrosis in the duodenal wall, and tissue infiltration by lymphocytes

Mucosal regeneration with restorative reaction findings in the duodenal wall associated with suture, trans-mural inflammation, normal intestinal mucosa, absence of peritoneal inflammatory reaction, and absence of fistula were found in all rabbits (Figure 2); chronic-fibrous repair findings were observed in three rabbits (in one of them a 30 % decreased lumen diameter was observed; data not shown) and nine rabbits showed granulomatous and fibroblastic repair features. Some lymphocytic infiltrate was a recurrent finding in all cases. No sign of residual Bioglue^®^ was observed. No abnormalities were demonstrated in the liver and biliary tract samples.

## DISCUSSION

Isolated duodenal injury is an operative finding in 0.2-3.7% of laparotomies performed for abdominal trauma and constitutes a major challenge for the surgeon^7^. When duodenal injury is confirmed, the surgeon must choose shortly an appropriate method of repair^14^. A number of treatment options have been described that indicates lack of satisfaction with the proposed procedures for duodenal repair^24^. Most of duodenal injuries require a simple repair and only a small number of these needs a mayor surgical effort (even the Whipple procedure accompanied by drainage suction, GI restriction, parental nutrition and octreotide supplementation)^6^. Grade III duodenal lesions, however, require a complex repair or duodenal decompression^1^ ^,^ ^16^. A complex repair has two major disadvantages: it is time consuming and technically demanding. Mortality rate of duodenal injury is 12% but range from 5-25%, largely due to associated injuries^8^ ^,^ ^14^ ^,^ ^24^ ^,^ ^26^. 

We tested a procedure in an animal model that can reduce morbidity and mortality of grade III duodenal lesions, i.e., parietal peritoneum grafting with or without Bioglue^®^ support. As a whole, in this study, we chose parietal peritoneum as a graft because: a) mesothelium in its surface has the same origin as the duodenal peritoneum and muscle layer; b) it is readily available and does not need any additional incisions; and c) it is readily available in large quantities.

Autologous peritoneum is an interesting material to be tried for intestine grafting because the ability of regeneration and trans-differentiation of mesothelial cells. Its mesothelium originates embryologically from the same stem cell as the intestine wall, i.e., mesenchyme stem cells originated from splacnopleura of the lateral mesoderm that differentiate into the serosa-muscle layers of intestine. They are also involved in the repair of the peritoneum damage following surgery or peritonitis. Mesothelial cells produce several cytokines, growth factors and extracellular matrix components, possessing anti-inflammatory and immunomodulatory properties. 

Carrel^9^ first described using peritoneum as vascular patch in 1901.A similar peritoneal graft was previously described in Japan^4^ ^,^ ^27^ and also in Mexico to treat vascular lesions^15^ and bile duct reconstruction^20^. When tissue samples were analyzed six months later, integrity of the intestinal wall was observed in both groups showing a smooth muscle layer and duodenal mucosa with thin compact villi and occasional lymphocyte infiltration. 

Peritoneum grafts appear to be safe, effective, easy to obtain, and cheap for repairing partial duodenal defects. Our results after six months showed that grafts were well integrated (although one of the reconstructions was somehow stenotic). It is necessary to highlight the low frequency of duodenum stenosis, especially because the proportion of circumference lost. Interestingly, the animals did not receive any postoperative antibiotics, octreotide, or other supplementary drugs^19^. 

It is unbelievable for us that, having already been widely demonstrated the usefulness of graft in several studies of venous reconstruction surgeons still do not recognize a role of graft in duodenal reconstruction. A similar falciform ligament graft has been described and might also be considered for reconstruction of duodenum or portal vein-superior mesenteric vein in the absence of synthetic graft^28^.

## CONCLUSION

Peritoneum auto-graft is an accessible and safe substitute for reconstruction of the grade III duodenal lesions with satisfactory results.
